# Biallelic loss‐of‐function variants in *RBL2* in siblings with a neurodevelopmental disorder

**DOI:** 10.1002/acn3.50992

**Published:** 2020-02-27

**Authors:** Theresa Brunet, Milena Radivojkov‐Blagojevic, Peter Lichtner, Verena Kraus, Thomas Meitinger, Matias Wagner

**Affiliations:** ^1^ Institute of Human Genetics, Faculty of Medicine Technical University Munich Munich Germany; ^2^ Institute of Human Genetics Helmholtz Zentrum München Neuherberg Germany; ^3^ Department of Pediatrics Klinik für Kinder‐ und Jugendmedizin München Klinik Schwabing und Harlaching Klinikum Rechts der Isar der Technischen Universität Munich Munich Germany; ^4^ Institute for Neurogenomics Helmholtz Zentrum München Neuherberg Germany

## Abstract

The *RBL2* locus has been associated with intelligence and educational attainment but not with a monogenic disorder to date. *RBL2* encodes p130, a member of the retinoblastoma protein family, which is involved in mediating neuron survival and death. Previous studies on p130 knockout mice revealing embryonic death and impaired neurogenesis underscore the importance of *RBL2* in brain development. Exome sequencing in two siblings with severe intellectual disability, stereotypies and dysmorphic features identified biallelic loss‐of‐function variants c.556C>T, p.(Arg186Ter) and a deletion of exon 13–17 in *RBL2* (NM_005611.3), establishing *RBL2* as a candidate gene for an autosomal recessive neurodevelopmental disorder.

## Introduction

Genome‐wide association studies (GWAS) have identified polymorphisms in the *RBL2* locus to be associated with intelligence and educational attainment,^1^, ^2^, ^3^ but the gene has not been described in a monogenic context to date.

**Figure 1:**
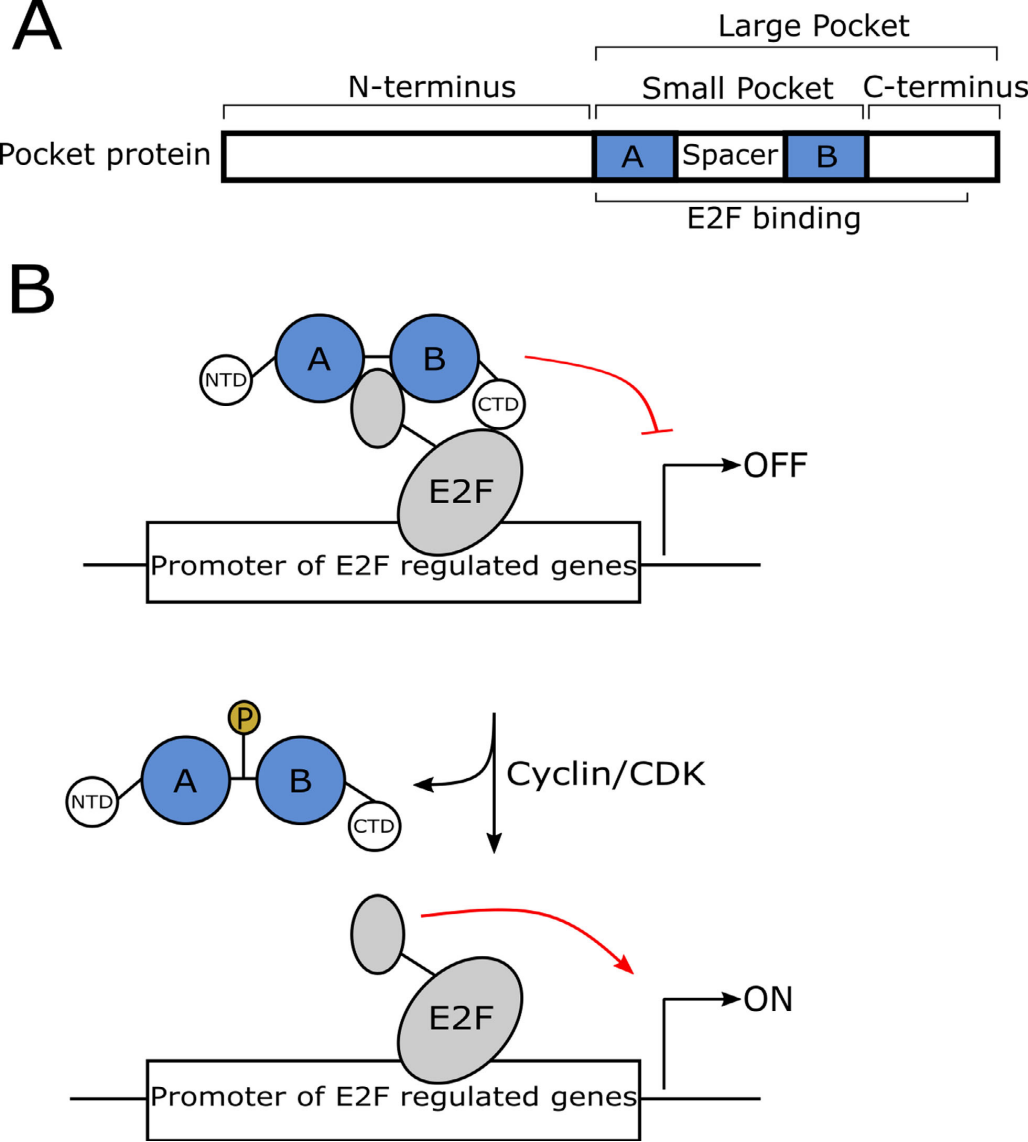
Structure of RB‐family proteins and model of cell‐cycle entry control by pocket proteins. (A) All three RB‐family proteins share large regions of homology, in particular the characteristic pocket domain. The small pocket region is comprised of an A‐box portion, a spacer domain and a B‐box portion. The large pocket region consists of the small pocket and the C‐terminal domain (adapted from Henley et al.)^9^. (B) In growth arrested cells, RB proteins repress transcriptional activity of E2F. Upon stimulation of cell cycle cyclin, dependent kinases become activated and phosphorylate RB family proteins. Hyperphosphorylated RB proteins dissociate from E2F, allowing E2F to recruit transcriptional coactivators and to relieve transcriptional repression (adapted from Sengupta et al.).^8^

*RBL2* [MIM: *180203] spans 22 exons and encodes RBL2/p130 (RB Transcriptional Corepressor Like 2), that – along with the related proteins RB1/p105 and RBL1/p107 – forms the retinoblastoma protein (RB) family.^4^ All three members share large regions of homology, in particular in the characteristic pocket domain comprised by an A‐box portion, a spacer domain, and a B‐box portion (schematized in Fig. 1A).^5^, ^6^ The RB family of proteins mediates gene expression and regulates the cell cycle by directly binding to the transactivation domain of E2F transcription factors as visualized in Figure 1B and by recruiting chromatin‐ and histone‐modifying enzymes.^7^, ^8^, ^9^ p130 is the preferred major binding partner and repressor of the transcription factor E2F4 and the predominant pocket protein in neurons. It is upregulated during neuronal differentiation and brain development.^10^, ^11^, ^12^, ^13^ A siRNA knockdown of p130 in rats induces death of cortical neurons.^10^ In a mouse model, homozygotes for a null allele display strain‐dependent embryonic lethality, impaired neurogenesis and myogenesis.^14^

We present two siblings with a neurodevelopmental disorder hallmarked by severe intellectual disability, delayed psychomotor development, infantile hypotonia, stereotypies and dysmorphic features. Exome sequencing (ES) identified compound‐heterozygous loss‐of‐function variants in the candidate gene *RBL2* segregating within the family proposing *RBL2* as an excellent candidate gene for neurodevelopmental disorders.

## Patients and Methods

### Probands and samples

The legal guardians of the subjects included in the study gave written informed consent for the collection and storage of clinical data, blood samples, photographs, and publication. This study was conducted according to the Declaration of Helsinki (2013) and approved by the local ethics committee of the Technical University Munich (#5360/12S).

### Exome sequencing

ES was performed for both affected siblings and their parents using a SureSelect *Human All Exon Kit* 60Mb, V6 (Agilent, Santa Clara, CA) for exome enrichment. Libraries were sequenced on an *Illumina NovaSeq6000* system (Illumina, San Diego, CA). Reads were aligned to the University of California Santa Cruz (UCSC) human reference assembly (hg19) with Burrows‐Wheeler Aligner (BWA, v.0.5.8).^15^ More than 96% of the exome was covered at least 20‐fold and average coverage was more than 93‐fold in both siblings. Single‐nucleotide variants and small insertions and deletions were detected using SAMtools v.0.1.7.^16^ Copy number variations were called with the software ExomeDepth.^17^ Variants were prioritized based on autosomal recessive (minor allele frequency, MAF < 0.1%).

### Haplotype analysis

Haplotype analysis was performed for all four siblings (two affected and two unaffected) and their parents using Infinium Global Screening Array‐24 v1.0 (Illumina, San Diego). Genotypes were called and exported with Genome Studio 2.0 (Illumina, San Diego). Variants with missing data and Mendelian errors were removed as well as uninformative variants using PLINK 1.9.^18^ Haplotypes were estimated with MERLIN using the "‐‐best" command.^19^

## Results

### Clinical findings

#### Patient II:1

**Figure 2:**
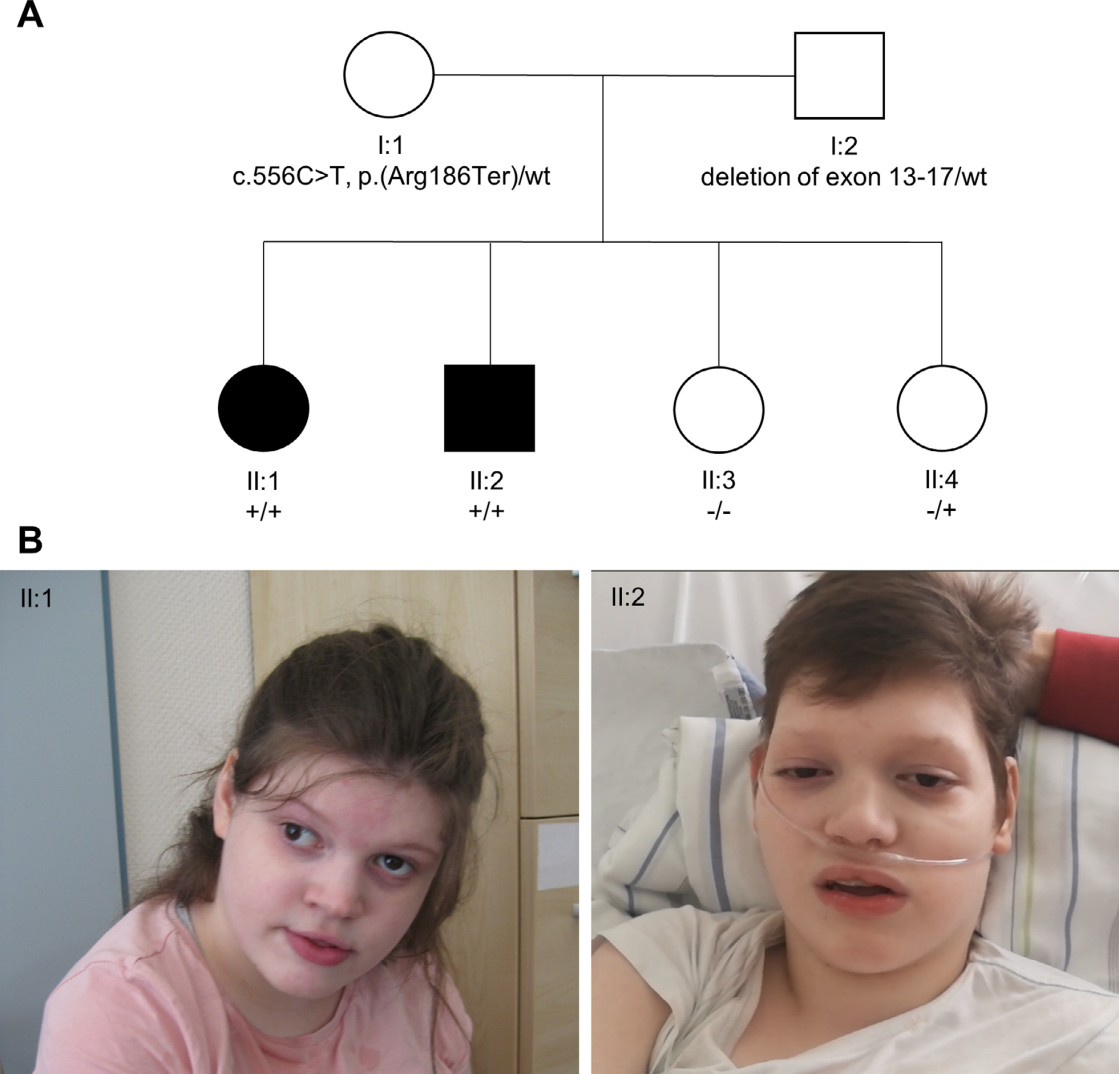
Family pedigree and photographs of both affected siblings. (A) Family pedigree demonstrates the inheritance of the identified null variants in *RBL2* (NM_005611.3) segregating within the family. (B) Photographs of individual II:1 and II:2 illustrate overlapping dysmorphic facial features (II:1: Low anterior hairline, round face, thin eyebrows, periorbital fullness, wide nasal bridge, full lips; II:2: Round face, sparse eyebrows, periorbital fullness, wide nasal bridge, full lips).

**Figure 3:**
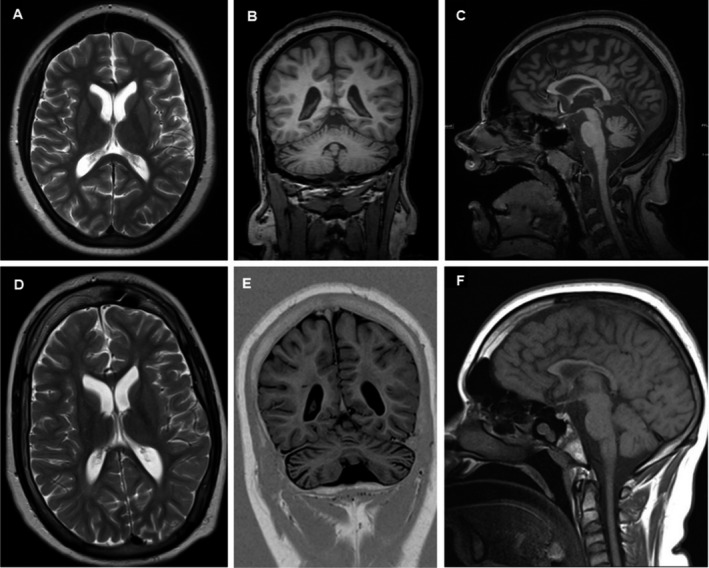
Brain MRI of individual II:1 (A–C) and individual II:2 (D–F). (A) Axial T2‐weighted, (B) coronal T1‐weighted TFE and (C) sagittal T1‐weighted TFE MRIs demonstrating supra‐ and infratentorial atrophy and hyperostosis of the skull. (D) Axial T2‐weighted, (E) coronal T1‐weighted inversion recovery, and (F) sagittal T1‐weighted SE MRIs demonstrating supra‐ and infratentorial atrophy, a thin corpus callosum and hyperostosis of the skull.

Patient II:1 was the first‐born female child of healthy and cognitively normal, nonconsanguineous parents (Fig. 2A). Pregnancy and birth were unremarkable. She was born late term with birth measurements within the normal range. After birth, she was noted to be floppy and global development delay was present early on. She learned to sit without support at 14 months and started to crawl at 20 months. Neither the ability to stand/walk without assistance nor an expressive language was ever acquired. Stereotypies as well as autoaggressive behavior were noted at an early stage. Strabismus divergens and horizontal gaze nystagmus were noted. Brain Magnetic Resonance Imaging (MRI) at the age of 17 years 10 months revealed supra‐ and infratentorial atrophy and hyperostosis of the skull (Fig. 3A–C). Electroencephalography (EEG) did not show any abnormalities and an epileptic event was never observed. When she first presented to our clinic at the age of 17 years, minor dysmorphic features (full lips, hypertelorism, thin eyebrows) very similar to her brother (Patient II:2) were present (Fig. 2B).

#### Patient II:2

Individual II:2 is the younger brother of individual II:1 (Fig. 2A) and was born late term with birth measurements within the normal range. After birth, he was noted to be floppy. Similar to his sister, he had severe global developmental delay. He was able to crawl at 24 months and learned to sit without assistance at 33 months. Standing/walking without support as well as expressive language were never developed and he did not acquire any communication/social skills. He showed stereotypic and autoaggressive behavior as well. In contrast to his sister, he displayed myoclonic seizures since the age of 10 years. Multiple antiepileptic trials did not result in seizure control. With the onset of the seizures, a first regression of his global development was noted that deteriorated after the occurrence of pneumonia at the age of 15 years. At the latest assessment at the age of 15 years 7 months, he had no head control and had lost the ability to sit. Brain MRI revealed supra‐ and infratentorial atrophy, a thin corpus callosum and hyperostosis of the skull (Fig. 3D–F). Besides a horizontal nystagmus, bilateral optic atrophy was observed. EEG showed a generalized background slowing with an additional intermittent slowing with spikes. He shared similar facial features with his sister (Fig. 2B). Both siblings did not receive any special education and were cared for at home by their parents.

Phenotypic details are summarized in Table S1.

### Molecular findings

ES identified compound‐heterozygous variants in *RBL2* (NM_005611.3) as the sole candidate in both affected individuals: A maternally inherited heterozygous nonsense variant c.556C>T, p.(Arg186Ter) and a paternally inherited heterozygous in‐frame deletion of exon 13–17 (of 22 exons in total) (Figs. S1 and S2). A list of biallelic variants can be found in Table S2. The nonsense variant is located in exon 3 (chr16:53476754) and is predicted to cause a premature termination of translation. In >18,000 exome datasets of our in‐house database, the variant was found once in a heterozygous, but not in a homozygous state. The Genome Aggregation Database (gnomAD) does not list the variant in a heterozygous or homozygous state.^20^ The heterozygous in‐frame deletion of exon 13–17 spans at least 5.479 bp (chr16:53499350–53504833) and is predicted to lead to a deletion of 335 amino acids (coding numbers 567–901) in the highly conserved pocket domain at the C‐terminal end of p130. The deletion is absent from our in‐house database as well as from gnomAD and is not listed in the Database of Genomic Variants (DGV). Due to their predicted loss‐of‐function character, the compound‐heterozygous variants were interpreted as likely disease causing. Subsequent haplotype analysis, performed on all four (two affected and two unaffected) siblings and their parents, revealed that the two disease haplotypes segregated in the family (Fig. 2A, Table S3).

### Minimal lifetime risk of RBL2‐associated disease

We extracted the combined minor allele frequency of loss‐of‐function alleles in gnomAD, which was 0.00027, to assess the minimal lifetime risk of *RBL2*‐associated disease. 20 As the frequency of biallelic carriers equals the square of the combined allele frequency under the assumption of Hardy–Weinberg equilibrium and mutual independence of these rare variants, 1 in 13.2 million newborns will have biallelic loss‐of‐function variants in *RBL2* and presumably be affected by the associated developmental disorder (Table S4).

## Discussion

*RBL2* has mainly been recognized due to its role in cancer etiology.^21^, ^22^ However, p130, which mediates gene expression by direct binding to the transcription factor E2F, is also a key regulator of maintaining neuron survival among the Rb family members.^10^ Equally important, p130, particularly expressed in basal progenitors, is involved in producing and differentiating neurons.^23^ Interestingly, RBL2 is not completely coexpressed with E2F4 (as well as RB1 and RBL1) indicating that the gene might have an independent, to date unknown, function in neurodevelopment.^13^ *RBL2* has not been linked to a monogenic disorder yet.

In both affected individuals, compound‐heterozygous null variants in *RBL2* (multi‐exon deletion and nonsense variant) were identified. The nonsense variant has a CADD score of 37 indicating a deleterious effect. The hypothesis of *RBL2* as a disease gene is further strengthened by the gnomAD gene constraint metrics showing a depletion of loss‐of‐function variants (ratio observed/expected count of LoF variants in *RBL2* = 0.15 (90% CI: 0.09–0.27)). In addition, there are no homozygous null variants in *RBL2* within the gnomAD dataset.^20^ This observation aligns with the mouse model, where p130 knockout resulted in strain‐dependent embryonic lethality and in impaired neurogenesis. Here, p130^−/−^ embryos exhibited growth arrest and died within the embryonic period. Besides a decreased number of differentiated myocytes within the myotome, immunohistochemistry demonstrated a reduced amount of motor neurons in the spinal cord and sensory neurons in the dorsal root ganglia, which could be a correlate to the muscular hypotonia observed in our cases. Furthermore, extensive apoptotic bodies in the neural tube, the brain and the dermomyotome were present. In addition, p130 knockout mice displayed an abnormally thin myocardium.^14^ Of note, neither individual II:1 nor individual II:2 had a congenital heart defect or signs of cardiomyopathy. siRNA‐mediated downregulation of p130 or E2F4 in rats resulted in death of cortical neurons.^10^ This observation was consistent with a previous work that showed an upregulation of p130 during brain development and neuronal differentiation in P19 cells.^12^

Taking its crucial role in neurogenesis into account, *RBL2* is an excellent candidate in the context of neurodevelopmental disorders. The affected siblings share the same main characteristics hallmarked by severe intellectual disability, delayed psychomotor development, infantile hypotonia, stereotypies, and minor dysmorphic features. Interestingly, the younger brother (individual II:2) appears to be more severely affected (optic atrophy, seizures and thin corpus callosum only in individual II:2) indicating that intrafamilial (and interfamilial) variability might be significant. In contrast to his sister, his condition has gradually deteriorated since the age of 10 years with the beginning of seizures. Recently, it was proposed that E2F4‐p130 complexes have a protective role in neurons from pathologic stresses, evoked by DNA damage and hypoxia.^24^ One could speculate that the seizures in individual II:2 may have acted as a trigger for neuronal death at some point.

Interestingly, the *RBL2* locus has been associated with intelligence and cognitive function further strengthening its role in brain development.^1^, ^2^, ^3^ Our findings establish *RBL2* as a candidate gene for an autosomal recessive neurodevelopmental disorder with intellectual disability, stereotypies and dysmorphic features adding variants with a monogenic effect to the allelic series. Besides the identification of additional patients with biallelic loss‐of‐function variants in *RBL2* and overlapping phenotypic characteristics, comprehensive functional studies to unmask the role of *RBL2* in neurodevelopmental disorders are essential to establish *RBL2* as a disease gene.

## Conflict of Interest

The authors declare that they have no conflict of interest related to the content of this article.

## Author Contribution

TB drafted the manuscript and was involved in genetic data analysis and genetic counseling of both patients. MW proposed and supervised the manuscript. MW, TM, MRB and PL contributed to the interpretation of genetic data. VK contributed to clinical data acquisition and was involved in the clinical management of both patients. All authors read and approved the manuscript before submission.

## Supporting information


**Figure S1.** Visualization of the maternally inherited variant in *RBL2*.
**Figure S2.** Visualization of paternally inherited variant in *RBL2*.
**Table S1.** Main phenotypic characteristics of the affected siblings with variants in *RBL2*.
**Table S2.** Listing of all biallelic variants identified in both affected siblings by exome sequencing.
**Table S3.** Haplotype analysis for all family members demonstrates segregation of the disease haplotypes with the disease. A represents the allele with the deletion of Exon 13–17; C represents the allele with the variant c.556C>T, p.(Arg186Ter).
**Table S4.** Assessment of the minimal lifetime risk of *RBL2*‐associated disease based on loss‐of‐function variants in gnomAD. The table shows all loss‐of‐function variants in *RBL2* with their respective allele frequency in the gnomAD v2.1.1 dataset.
