# Supplementary material for: Biallelic loss‐of‐function variants in RBL2 in siblings with a neurodevelopmental disorder
Source: Ann Clin Transl Neurol. 2020 Feb 27;7(3):390–6. doi: 10.1002/acn3.50992 (PMC7086002; doi:10.1002/acn3.50992)
Supplement: Supplementary file 1 — Figure S1. Visualization of the maternally inherited variant in RBL2. Figure S2. Visualization of paternally inherited variant in RBL2. Table S1. Main phenotypic characteristics of the affected siblings with variants in RBL2. Table S2. Listing of all biallelic variants identified in both affected siblings by exome sequencing. Table S3. Haplotype analysis for all family members demonstrates segregation of the disease haplotypes with the disease. A represents the allele with the deletion of Exon 13–17; C represents the allele with the variant c.556C>T, p.(Arg186Ter). Table S4. Assessment of the minimal lifetime risk of RBL2‐associated disease based on loss‐of‐function variants in gnomAD. The table shows all loss‐of‐function variants in RBL2 with their respective allele frequency in the gnomAD v2.1.1 dataset. [file ACN3-7-390-s001.docx]

**Supplemental information**

**Supplementary Figures**


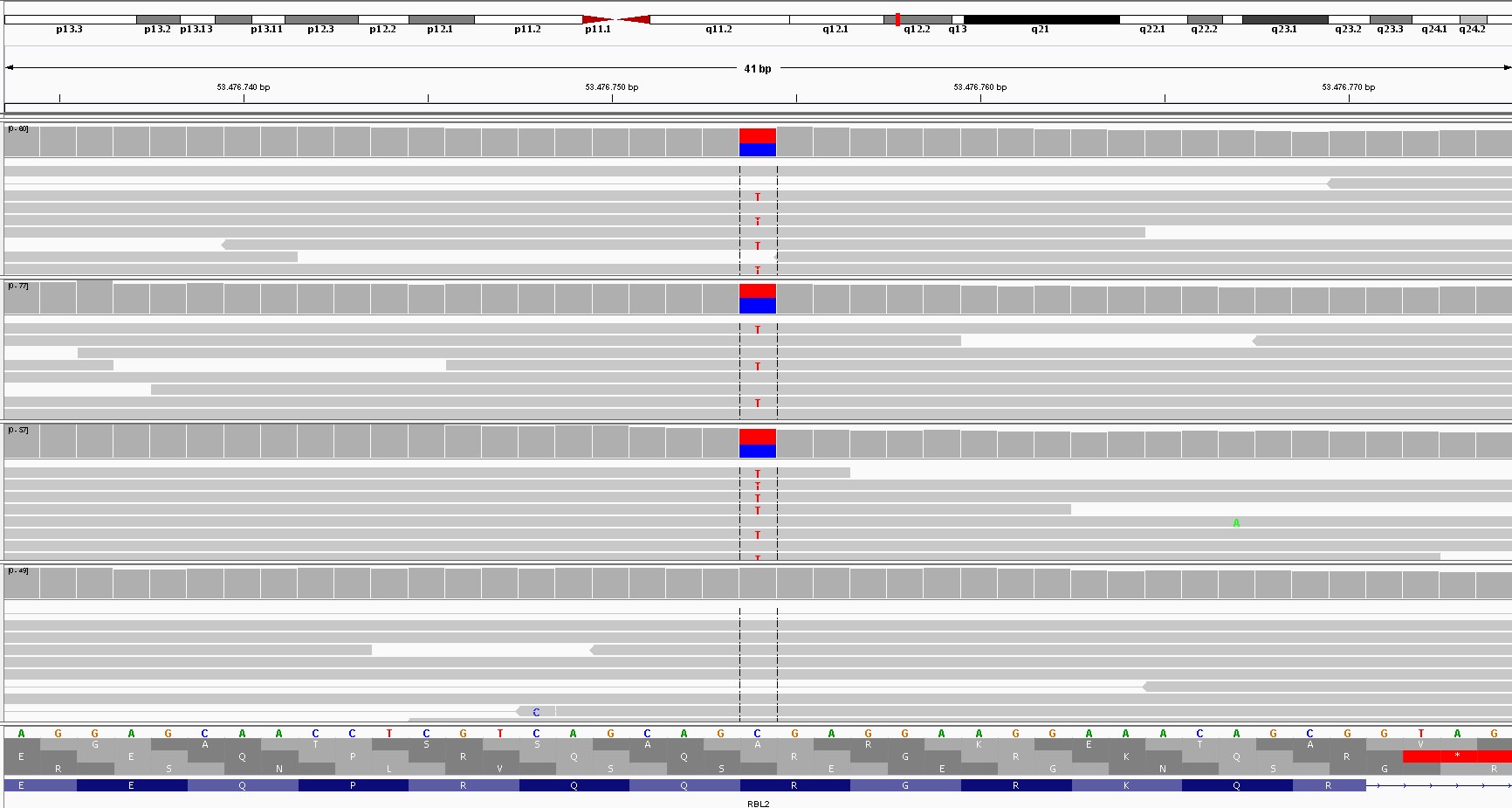


Individual II:1

Individual II:2

Mother I:1

Father I:2

**Supplementary Figure 1. Visualization of the maternally inherited variant in *RBL2*.** Illustration of the maternally inherited heterozygous nonsense variant NM_005611.3: c.556C>T, p.(Arg186Ter) in exon 3 of *RBL2* (chr16:53476754) in both affected individuals in the Integrative Genomics Viewer (IGV). In the top panel, the cytogenetic localization of *RBL2* (red line) is displayed on chromosome 16 (16q12.2). The bottom panel shows the reference sequence of *RBL2*. Grey bars represent next generation sequencing (NGS) reads aligned to the respective chromosomal region.


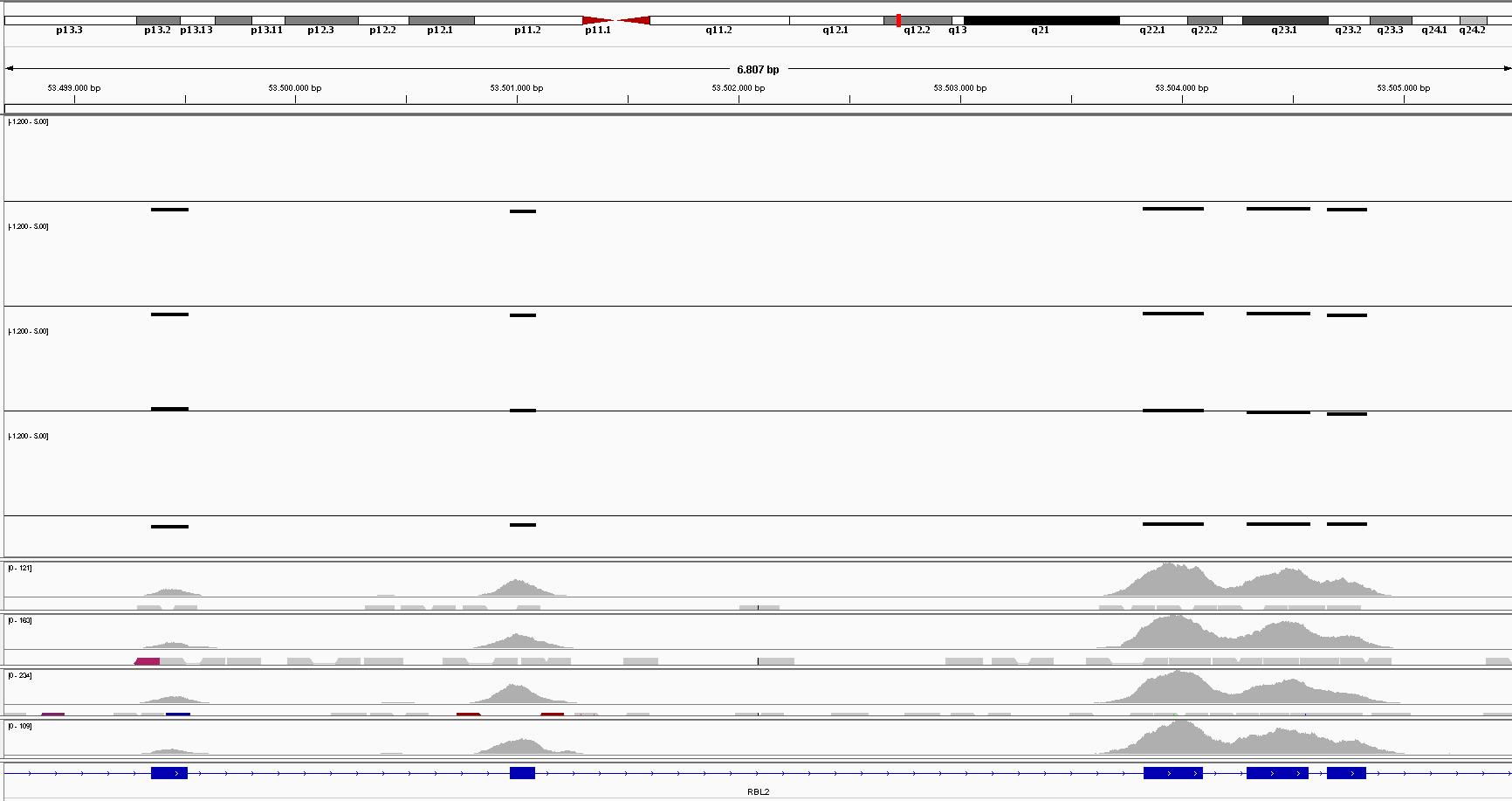


Individual II:1

Individual II:2

Mother I:1

Father I:2

Individual II:1

Individual II:2

Mother I:1

Father I:2

**Supplementary Figure 2. Visualization of paternally inherited variant in *RBL2*.** Illustration of the paternally inherited heterozygous multi-exon deletion (exon 13-17) in the Integrative Genomics Viewer (IGV) as analysed using the program ExomeDepth. In the top panel, the cytogenetic localization of *RBL2* (red line) is displayed on chromosome 16 (16q12.2). The next four panels indicate the relative read depth at the respective exon for both affected siblings and the parents. The thin line represents the averaged read depth of 100 control exome sequencing datasets. The bold lines indicate that both affected individuals as well as the father have a reduced read depth across exon 13 – 17. The read depth was quantified with II:1 having 0.569, II:2 0.555 and I:2 0.536 of the control read depth indicating a heterozygous deletion. The bottom panel shows the exon-intron boundaries of *RBL2* with the boxes representing exons. Grey peaks indicate the NGS coverage at the respective location.

**Supplementary Tables:**

**Supplementary Table 1.** Main phenotypic characteristics of the affected siblings with variants in *RBL2*.

|  | Patient II:1 | Patient II:2 |
| --- | --- | --- |
| Gender | Female | Male |
| Pregnancy | Unremarkable | Unremarkable |
| Prematurity | No, vaginal delivery at 42 weeks | No, vaginal delivery at 42 weeks |
| Birth length | 3650g (52. P.) | 3850g (57. P.) |
| Birth weight | 56cm (94. P.) | 54cm (62. P.) |
| Head circumference | n.a. | n.a. |
| APGAR | 10/10. | n.a. |
| Last assessment |  |  |
| Age | 17 years 10 month | 15 years 7 months |
| Height | 150cm (<3.P.) | 165cm (9.P.) |
| Weight | 62kg (70.P.) | 60kg (39.P.) |
| OFC | 54cm (25.P.) | 55cm (21.P.) |
| Developmental milestones |  |  |
| Looking at and reaching for faces and toys | At 8 months | n.a. |
| Sitting without support | At 14 months | At 33 months |
| Rolling over | n.a. | At 8 months |
| Crawling around | At 20 months | At 24 months |
| Standing without assistance | No | No |
| Walking without assistance | No | No |
| Speech/language | Absent | Absent |
| Social interaction | Absent | Absent |
| Overall development delay | Yes, severe | Yes, severe |
| Intellectual disability | Yes, severe | Yes, severe |
| Developmental regression | No | Yes, with begin of seizures at age of 10 years |
| Neurological findings |  |  |
| Nystagmus | Yes | Yes |
| Muscle tone | Muscular hypotonia in early infancy (“floppy infant”) | Muscular hypotonia in early infancy (“floppy infant”) |
| Spasticity | No | Yes |
| Seizures | No | Yes, myoclonic seizures |
| EEG | No epileptic EEG-activities | Generalized background slowing with an additional intermittent slowing with spikes. |
| Stereotypies | Yes | Yes |
| Autoaggressive behavior | Yes | Yes |
| Visual | Strabismus divergens, horizontal gaze nystagmus | Bilateral optic atrophy, absent visual evoked potential (VEP), nystagmus |
| Auditory | n.a. | Absent auditory evoked potential (AEP) |
| Organ systems |  |  |
| Cardiovascular | ECG and echocardiogram: unremarkable | ECG and echocardiogram: unremarkable |
| Respiratory | Unremarkable | Pneumonia and mastoiditis with sepsis at age 15 years |
| Gastrointestinal | Unremarkable | Unremarkable |
| Dermatologic | Naevus flammeus at forehead | Unremarkable |
| Skeletal | hyperostosis of the skull | Myositis ossificans at right hip (radiation and subsequent operation), hyperostosis of the skull |
| Genitourinary | n.a. | Undescended left testis |
| Immunologic | n.a. | Selective immunoglobulin IgG2/IgG4/IgA deficiency |
| Others | Obesity | Tooth decay |
| Brain MRI | Supra- and infratentorial atrophy | Supra- and infratentorial atrophy, thin corpus callosum |
| Dysmorphic features | Low anterior hairline, round face, thin eyebrows, periorbital fullness, wide nasal bridge, bifid nasal tip, full lips | Round face, sparse eyebrows, periorbital fullness, wide nasal bridge, bifid nasal tip, full lips, high-arched palate |

ECG = electrocardiography, EEG = electroencephalography, MRI = magnetic resonance imaging, n.a. = not available

**Supplementary Table 2:** Listing of all biallelic variants identified in both affected siblings by exome sequencing.

| **Chr** | **Gene** | **Transcript** | **Variant** | **Omim** | **Function** | **pph2** | **Variant alleles** | **SNV Qual** | **Geno- type Qual** | **Map Qual** | **Depth** | **Percent Var** | **dbSNP 142** | **HGMD** | **ClinVar** | **1000 genomes AF** |
| --- | --- | --- | --- | --- | --- | --- | --- | --- | --- | --- | --- | --- | --- | --- | --- | --- |
| [chr13:84454391-84454391](http://genome-euro.ucsc.edu/cgi-bin/hgTracks?db=hg19&highlight=hg19.chr13:84454390-84454392&position=chr13:84454390-84454392) | [SLITRK1](https://evadb.helmholtz-muenchen.de/cgi-bin/mysql/snv-vcf/searchGene.pl?g.genesymbol=SLITRK1) | NM_052910.2 | c.1252A>T, p.Thr418Ser | [609678](http://www.ncbi.nlm.nih.gov/omim/609678) | missense | benign | 2 | 282 | 99 | 60 | 147 | 100 | [rs150504822](https://www.ncbi.nlm.nih.gov/SNP/snp_ref.cgi?type=rs&rs=rs150504822) | [CM1613961](http://ihgseq13.helmholtz-muenchen.de/hgmd/pro/mut.php?accession=CM1613961) |  | 0.000199681 |
| [chr16:2134508-2134508](http://genome-euro.ucsc.edu/cgi-bin/hgTracks?db=hg19&highlight=hg19.chr16:2134507-2134509&position=chr16:2134507-2134509) | [TSC2](https://evadb.helmholtz-muenchen.de/cgi-bin/mysql/snv-vcf/searchGene.pl?g.genesymbol=TSC2) | NM_000548.3 | c.4285G>T, p.Ala1429Ser | [191092](http://www.ncbi.nlm.nih.gov/omim/191092) | missense | benign | 1 | 225 | 99 | 60 | 222 | 52 | [rs45474795](https://www.ncbi.nlm.nih.gov/SNP/snp_ref.cgi?type=rs&rs=rs45474795) |  | [Benign/Likely_benign](https://www.ncbi.nlm.nih.gov/clinvar/?term=50180%5balleleid%5d) | 0.00139776 |
| [chr16:2134982-2134984](http://genome-euro.ucsc.edu/cgi-bin/hgTracks?db=hg19&highlight=hg19.chr16:2134981-2134985&position=chr16:2134981-2134985) |  |  | c.4527_4529delCTT, p.Phe1510del |  | indel |  | 1 | 217 | 99 | 59 | 481 | 49 |  | [CD962170](http://ihgseq13.helmholtz-muenchen.de/hgmd/pro/mut.php?accession=CD962170) | [Benign(8) Likely_benign(3) Uncertain_significance(1)](https://www.ncbi.nlm.nih.gov/clinvar/?term=58464%5balleleid%5d) | 0.00399361 |
| [chr1:152277890-152277890](http://genome-euro.ucsc.edu/cgi-bin/hgTracks?db=hg19&highlight=hg19.chr1:152277889-152277891&position=chr1:152277889-152277891) | [FLG](https://evadb.helmholtz-muenchen.de/cgi-bin/mysql/snv-vcf/searchGene.pl?g.genesymbol=FLG) | NM_002016.1 | c.9472T>C, p.Ser3158Pro | [135940](http://www.ncbi.nlm.nih.gov/omim/135940) | missense | possibly damaging | 1 | 185 | 99 | 28 | 74 | 49 |  |  |  | 0 |
| [chr1:152282635-152282635](http://genome-euro.ucsc.edu/cgi-bin/hgTracks?db=hg19&highlight=hg19.chr1:152282634-152282636&position=chr1:152282634-152282636) |  |  | c.4727T>A, p.Val1576Glu |  | missense | benign | 1 | 225 | 99 | 60 | 763 | 46 | [rs142456327](https://www.ncbi.nlm.nih.gov/SNP/snp_ref.cgi?type=rs&rs=rs142456327) |  |  | 0 |
| [chr1:156696974-156696974](http://genome-euro.ucsc.edu/cgi-bin/hgTracks?db=hg19&highlight=hg19.chr1:156696973-156696975&position=chr1:156696973-156696975) | [ISG20L2](https://evadb.helmholtz-muenchen.de/cgi-bin/mysql/snv-vcf/searchGene.pl?g.genesymbol=ISG20L2) | NM_030980.1 | c.471G>C, p.Gln157His |  | missense,regulation | benign | 1 | 225 | 99 | 60 | 80 | 56 | [rs138574933](https://www.ncbi.nlm.nih.gov/SNP/snp_ref.cgi?type=rs&rs=rs138574933) |  |  | 0.00119808 |
| [chr1:156697407-156697407](http://genome-euro.ucsc.edu/cgi-bin/hgTracks?db=hg19&highlight=hg19.chr1:156697406-156697408&position=chr1:156697406-156697408) |  |  | c.38C>A, p.Pro13His |  | missense,regulation | possibly damaging | 1 | 225 | 99 | 59 | 46 | 50 |  |  |  | 0 |
| [chr4:9213249-9213249](http://genome-euro.ucsc.edu/cgi-bin/hgTracks?db=hg19&highlight=hg19.chr4:9213248-9213250&position=chr4:9213248-9213250) | [USP17L10](https://evadb.helmholtz-muenchen.de/cgi-bin/mysql/snv-vcf/searchGene.pl?g.genesymbol=USP17L10) | NM_001256852.1 | c.867C>A, p.Asn289Lys |  | missense |  | 2 | 282 | 99 | 45 | 112 | 100 |  |  |  | 0 |
| [chr4:9213514-9213514](http://genome-euro.ucsc.edu/cgi-bin/hgTracks?db=hg19&highlight=hg19.chr4:9213513-9213515&position=chr4:9213513-9213515) |  |  | c.1132T>A, p.Trp378Arg |  | missense |  | 2 | 129 | 99 | 30 | 34 | 100 |  |  |  | 0 |

**Supplementary Table 3.** Haplotype analysis for all family members demonstrates segregation of the disease haplotypes with the disease. A represents the allele with the deletion of Exon 13 -17; C represents the allele with the variant c.556C>T, p.(Arg186Ter).

| **SNP** | **Chromosome** | **Position** | **I:1** | **I:2** | **II:1** | **II:2** | **II:3** | **II:4** |
| --- | --- | --- | --- | --- | --- | --- | --- | --- |
| rs7204230 | 16 | 53.192.331 | C : D | A : B | C \| A | C \| A | D \| B | D \| A |
| rs1073454 | 16 | 53.196.042 | C : D | A : B | C \| A | C \| A | D \| B | D \| A |
| rs62049787 | 16 | 53.318.263 | C : D | A : B | C \| A | C \| A | D \| B | D \| A |
| GSA-rs72799639 | 16 | 53.321.358 | C : D | A : B | C \| A | C \| A | D \| B | D \| A |
| GSA-rs3743771 | 16 | 53.358.439 | C : D | A : B | C \| A | C \| A | D \| B | D \| A |
| rs7191257 | 16 | 53.363.301 | C : D | A : B | C \| A | C \| A | D \| B | D \| A |
| rs8052485 | 16 | 53.376.791 | C : D | A : B | C : A | C : A | D : B | D : A |
| rs3095574 | 16 | 53.416.622 | C : D | A : B | C \| A | C \| A | D \| B | D \| A |
| rs34425733 | 16 | 53.427.828 | C : D | A : B | C \| A | C \| A | D \| B | D \| A |
| rs12444248 | 16 | 53.430.300 | C : D | A : B | C \| A | C \| A | D \| B | D \| A |
| rs17800727 | 16 | 53.481.010 | C : D | A : B | C \| A | C \| A | D \| B | D \| A |
| rs13332406 | 16 | 53.489.705 | C : D | A : B | C \| A | C \| A | D \| B | D \| A |
| deletion beginning | 16 | 53.499.350 |  |  |  |  |  |  |
| deletion ending | 16 | 53.504.833 |  |  |  |  |  |  |
| GSA-rs3809634 | 16 | 53.538.157 | C : D | A : B | C \| A | C \| A | D \| B | D \| A |
| rs3095571 | 16 | 53.544.993 | C : D | A : B | C \| A | C \| A | D \| B | D \| A |
| rs3095631 | 16 | 53.545.800 | C : D | A : B | C \| A | C \| A | D \| B | D \| A |
| rs17194040 | 16 | 53.550.898 | C : D | A : B | C \| A | C \| A | D \| B | D \| A |
| rs76334525 | 16 | 53.552.436 | C : D | A : B | C \| A | C \| A | D \| B | D \| A |
| rs9788828 | 16 | 53.577.519 | C : D | A : B | C \| A | C \| A | D \| B | D \| A |
| rs16952304 | 16 | 53.578.713 | C : D | A : B | C \| A | C \| A | D \| B | D \| A |
| GSA-rs7193898 | 16 | 53.584.921 | C : D | A : B | C \| A | C \| A | D \| B | D \| A |
| rs1362572 | 16 | 53.587.920 | C : D | A : B | C \| A | C \| A | D \| B | D \| A |

**Supplementary Table 4.** Assessment of the minimal lifetime risk of *RBL2*-associated disease based on loss-of-function variants in gnomAD. The table shows all loss-of-function variants in *RBL2* with their respective allele frequency in the gnomAD v2.1.1 dataset.

| **Chromosome** | **Position** | **rsID** | **Reference** | **Alternate** | **Consequence** | **Transcript Consequence** | **Annotation** | **Allele Count** | **Allele Number** | **Allele Frequency** |
| --- | --- | --- | --- | --- | --- | --- | --- | --- | --- | --- |
| 16 | 53468550 | rs893977286 | GAC | G | p.Asp28GlyfsTer55 | c.83_84delAC | frameshift_variant | 1 | 31174 | 3,2078E-05 |
| 16 | 53468553 | rs577332958 | GGCGAGGCGGAAGA | G | p.Gly29AlafsTer41 | c.86_98delGCGAGGCGGAAGA | frameshift_variant | 1 | 31256 | 3,1994E-05 |
| 16 | 53472953 | rs1211544318 | G | GTGCCTTA | p.Tyr92CysfsTer27 | c.268_274dupGCCTTAT | frameshift_variant | 1 | 242584 | 4,1223E-06 |
| 16 | 53485741 | rs1396325692 | AAGGT | A | c.766+3_766+6delAGGT | c.766+3_766+6delAGGT | splice_donor_variant | 1 | 251304 | 3,9792E-06 |
| 16 | 53485744 | rs1360109091 | G | A | c.766+1G>A | c.766+1G>A | splice_donor_variant | 1 | 31402 | 3,1845E-05 |
| 16 | 53487515 | rs746503944 | T | A | p.Tyr306Ter | c.918T>A | stop_gained | 5 | 242100 | 2,0653E-05 |
| 16 | 53487525 | rs777143220 | GT | G | c.927+4delT | c.927+4delT | splice_donor_variant | 4 | 246320 | 1,6239E-05 |
| 16 | 53487644 | rs369293435 | AC | A | p.Thr319MetfsTer4 | c.956delC | frameshift_variant | 1 | 248952 | 4,0168E-06 |
| 16 | 53488695 | rs1392685802 | G | T | p.Gly374Ter | c.1120G>T | stop_gained | 1 | 251200 | 3,9809E-06 |
| 16 | 53488699 | rs1349540723 | CAG | C | p.Glu376AspfsTer3 | c.1128_1129delGA | frameshift_variant | 1 | 251190 | 3,9811E-06 |
| 16 | 53488752 | rs755985698 | AAGGTG | A | c.1179+1_1179+5delGTGAG | c.1179+1_1179+5delGTGAG | splice_donor_variant | 3 | 246678 | 1,2162E-05 |
| 16 | 53493444 | rs1422680674 | A | AG | p.Thr421SerfsTer5 | c.1261_1262insG | frameshift_variant | 1 | 249358 | 4,0103E-06 |
| 16 | 53493509 | rs770275673 | GA | G | p.Lys443AsnfsTer21 | c.1329delA | frameshift_variant | 1 | 208972 | 4,7853E-06 |
| 16 | 53493529 | rs1219562068 | GGTTA | G | c.1346+4_1346+7delAGTT | c.1346+4_1346+7delAGTT | splice_donor_variant | 1 | 31376 | 3,1871E-05 |
| 16 | 53495651 | rs771449976 | A | G | c.1347-2A>G | c.1347-2A>G | splice_acceptor_variant | 1 | 217786 | 4,5917E-06 |
| 16 | 53496535 | rs868490573 | G | T | p.Glu510Ter | c.1528G>T | stop_gained | 1 | 212680 | 4,7019E-06 |
| 16 | 53498136 | rs762145795 | A | C | c.1561-2A>C | c.1561-2A>C | splice_acceptor_variant | 1 | 213036 | 4,694E-06 |
| 16 | 53498167 | rs757065708 | ATC | A | p.Leu533GlyfsTer5 | c.1596_1597delCT | frameshift_variant | 1 | 241176 | 4,1463E-06 |
| 16 | 53498206 | rs780168032 | TTA | T | p.Tyr544Ter | c.1632_1633delTA | frameshift_variant | 1 | 250536 | 3,9914E-06 |
| 16 | 53504347 | rs1340788698 | TG | T | p.Gln769ArgfsTer5 | c.2304delG | frameshift_variant | 1 | 251100 | 3,9825E-06 |
| 16 | 53504657 | rs756889127 | G | C | c.2527-1G>C | c.2527-1G>C | splice_acceptor_variant | 4 | 250774 | 1,5951E-05 |
| 16 | 53504700 | rs772388520 | A | T | p.Lys857Ter | c.2569A>T | stop_gained | 1 | 251256 | 3,98E-06 |
| 16 | 53513090 | rs1437736636 | CAG | C | p.Asn911HisfsTer6 | c.2730_2731delGA | frameshift_variant | 2 | 282342 | 7,0836E-06 |
| 16 | 53513867 | rs1360758212 | CA | C | p.His949LeufsTer8 | c.2846delA | frameshift_variant | 2 | 248692 | 8,0421E-06 |
| 16 | 53515637 | rs781142746 | C | T | p.Arg1047Ter | c.3139C>T | stop_gained | 1 | 251232 | 3,9804E-06 |
| 16 | 53524093 | rs749728758 | AAG | A | p.Gly1103AsnfsTer8 | c.3306_3307delAG | frameshift_variant | 1 | 250370 | 3,9941E-06 |
|  |  |  |  |  |  |  |  |  |  |  |
|  |  |  |  |  |  |  |  |  | **Combined allele frequency** | 0,00027486 |
|  |  |  |  |  |  |  |  |  | **minimal lifetime risk** | 7,5546E-08 |
